# Generalization of the Conformity Index for Multi-Target Radiotherapy Plans

**DOI:** 10.3390/cancers18030426

**Published:** 2026-01-28

**Authors:** Yong Sang, Jun Dang, Jianan Wu, Yanling Wu, Enzhuo Quan, Jianrong Dai

**Affiliations:** 1 Department of Radiation Oncology, National Cancer Center/National Clinical Research Center for Cancer/Cancer Hospital & Shenzhen Hospital, Chinese Academy of Medical Sciences and Peking Union Medical College, Shenzhen 518116, China; sangyong@cicams-sz.org.cn (Y.S.);; 2 Department of Radiation Oncology, National Cancer Center/National Clinical Research Center for Cancer/Cancer Hospital, Chinese Academy of Medical Sciences and Peking Union Medical College, Beijing 100021, China

**Keywords:** radiotherapy planning, generalized conformity index, evaluation, multi-target plans

## Abstract

**Background and Purpose:** Based on the distortion of the current conformity index (CI) formula in handling multi-target plans, the $V_{TV}$ parameter in the current CI formula has been redefined to more accurately calculate the CI value of multi-target plans, providing a reference for clinical applications. **Methods and Materials:** Considering the limitations of the current $V_{TV}$ calculation formula in CI proposed by van’t Riet and Paddick, a new $V_{TV}$ has been defined to better reflect the true conformity of the target volume in multi-target planning. We selected 15 breast cancer (BC) plans with PTVsc and PTVcw as the target volumes, and 15 nasopharyngeal carcinoma (NPC) plans with PTVp, PTVn, PTVrpn, PTV1, and PTV2 as target volumes. $V_{TV}^{new}$ and $CI_{new}$ were calculated using the proposed formulas, while $V_{TV}^{old}$ and $CI_{old}$ were calculated using traditional formulas based on van’t Riet and Paddick. A paired, two-tailed Wilcoxon signed-rank test was conducted to compare $V_{TV}^{new}$ with $V_{TV}^{old}$, and $CI_{new}$ with $CI_{old}$ across all target volumes. Pearson’s correlation analysis was performed between $CI_{new}$ and $CI_{old}$. **Results:** For BC, the $V_{TV}$ values calculated by the two methods for PTVsc and PTVcw showed statistically significant differences; the values calculated in this study were significantly lower than those calculated by van’t Riet and Paddick (*p* < 0.05). Consequently, the $CI_{new}$ values for BC were significantly higher than $CI_{old}$. These results were consistent with those for PTVp, PTVn, PTVrpn, and PTV2 in NPC. For PTV1 in NPC, the results calculated by the two formulas were identical. **Conclusions:** The new $V_{TV}$ calculation formula eliminates the influence of dose spillage from adjacent targets, retaining only the prescription dose range of the specific target under analysis. This makes the calculated CI more reflective of true conformity compared to traditional formulas. We recommend using the proposed formula to calculate CI values for multi-target plans such as those for BC and NPC.

## Simple Summary

This study proposes a generalized method to calculate the Conformity Index (CI) for multi-target radiotherapy plans (e.g., breast or nasopharyngeal cancer). Standard CI formulas are often distorted in these complex scenarios because they erroneously include dose spillover from adjacent targets. To address this, we redefined the Target Volume $\left(V_{TV}\right)$ parameter to mathematically isolate the prescription dose region of each specific target. Validation on clinical plans demonstrated that the new formula effectively eliminates interference from neighboring targets, providing CI values that accurately reflect the true dose conformity. This improved calculation is recommended for the objective evaluation of multi-target radiotherapy plans.

## 1. Introduction

Radiation therapy aims to deliver a curative prescription dose to the target volume while minimizing the dose delivered to organs at risk (OARs) and surrounding normal tissues. With the advancement of delivery technologies, Volumetric Modulated Arc Therapy (VMAT) has become a standard treatment modality for complex malignancies due to its ability to achieve highly conformal dose distributions [1,2,3]. A key advantage of VMAT is its ability to treat multiple targets simultaneously, such as in Simultaneous Integrated Boost (SIB) techniques. SIB offers significant biological and logistical advantages over sequential boost approaches by delivering different dose levels to distinct target volumes within a single fraction. This not only improves treatment efficiency by reducing overall treatment time but also enhances radiobiological effectiveness by delivering a higher dose per fraction to the gross tumor volume while simultaneously treating elective nodal regions [4,5].

The clinical efficacy of VMAT has been widely demonstrated across various tumor sites, including lung [1,2,3], esophageal [6,7], rectal [4,8], and brain cancers [9], as well as complex multi-target scenarios such as nasopharyngeal carcinoma (NPC) [5,10,11,12,13,14,15,16], breast cancer (BC) [17,18,19,20,21,22], and cervical cancer [23,24,25,26]. However, the increased complexity of these VMAT plans necessitates rigorous quality assurance to ensure that the delivered dose strictly matches the planning target volume (PTV). Therefore, evaluating the degree of conformity between the prescription dose and the PTV is essential. Robust evaluation indices help clinicians objectively quantify this matching degree, facilitating the selection of the optimal plan among competing alternatives [27,28,29].

The Conformity Index (CI) is one of the most widely established metrics for this purpose. It represents the degree of spatial overlap between the prescription isodose line and the PTV. CI extends standard Dose-Volume Histogram (DVH) analysis by providing a 3D geometric assessment of how well the dose distribution conforms to the size and shape of the target [29]. Beyond routine plan selection, CI is also critical for comparing different radiotherapy modalities, such as Gamma Knife, linear accelerators, and particle therapy [30].

Various formulas have been proposed to calculate CI values [28]. The most widely used definitions were proposed by van’t Riet [31] and Paddick [30]. While these traditional formulas perform well for single targets or multi-target cases with simple inclusion geometries (e.g., cervical cancer with lymph nodes), they often fail in modern multi-target VMAT plans characterized by complex geometric relationships. These relationships may include independent targets (e.g., distinct nodes in NPC) or partially overlapping targets with different dose levels (e.g., chest wall and supraclavicular nodes in BC) [30,31,32].

In such complex scenarios, the standard calculation of the Target Volume covered by the reference isodose $(V_{TV})\;$ becomes distorted. The traditional formula calculates the total volume of the prescription isodose line anywhere in the body, erroneously including dose spillage from adjacent targets. This leads to a distorted CI value that underestimates the plan’s true conformity. While previous attempts have utilized manual auxiliary structures to isolate target dose [33,34], a fully automated mathematical definition is needed. Recent studies have highlighted this limitation, noting that standard indices like the Paddick CI are heavily influenced by target number and dose bridging in single-isocenter multi-target plans [35].

To address this, we have redefined the $V_{TV}$ parameter in the standard CI formula. This generalization ensures that the CI value accurately reflects target conformity in multi-target planning by mathematically isolating the target of interest. We demonstrate the application of this generalized CI in VMAT plans for BC and NPC and compare it with the traditional van’t Riet and Paddick calculations.

## 2. Materials and Methods

### 2.1. Redefinition of the CI

The CI formula defined by van’t Riet and Paddick is:

(1) $$CI=\frac{TV_{PV}^{2}}{V_{PTV}\times V_{TV}}$$

where $TV_{PV}$ represents the target volume covered by the reference prescription isodose, $V_{PTV}$ represents the target volume, and $V_{TV}$ represents the total volume of the reference isodose.

**Figure 1:**
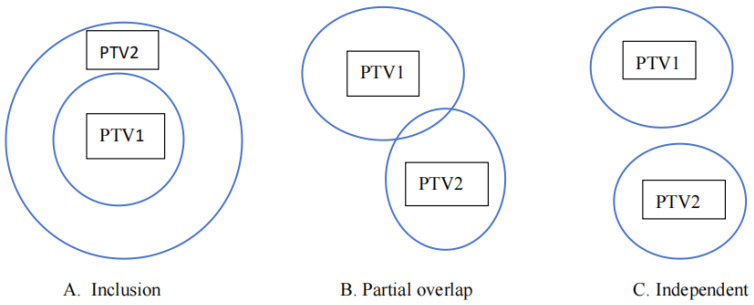
Three types of relationships between PTV1 and PTV2.

For a single-target plan, $V_{TV}$ is derived solely from the target area, making Formula (1) accurate. However, for a two-target plan (PTV1 and PTV2), three geometric relationships exist: inclusion, partial overlap, and complete independence (Figure 1). In cases of partial overlap or independence, the $V_{TV}$ for PTV1 calculated by the traditional formula may include the prescription dose volume generated for PTV2. This inclusion causes severe distortion in the CI calculation. This distortion exacerbates as the number of targets increases, as seen in NPC cases.

To eliminate the influence of other targets, we propose a redefined $V_{TV}$ (denoted as $V_{TV}^{new}$) for a specific target $PTV_{i}$:

(2) $$V_{TV}^{new}=(PTV_{i}^{ext}-\sum PTV_{higher}+PTV_{i})\cap V_{pd}$$

(3) $$PTV_{i}^{ext}=PTV_{i}+Expansion$$

where:

- $PTV_{i}^{ext}$ is the volumetric expansion of $PTV_{i}$ (0.5 cm Superior/Inferior, 1.0 cm Left/Right, 1.0 cm Anterior/Posterior). This expansion ensures the region of interest encompasses the likely prescription dose spillage for $PTV_{i}$.
- $PTV_{higher}$ represents the union of all other PTVs with prescription doses greater than or equal to that of $PTV_{i}$.
- $V_{pd}$ is the total volume covered by the prescription isodose for $PTV_{i}$.
- The Boolean operations (−) and (+) represent subtraction and union, respectively.
- The symbol ∩ represents the intersection.

By subtracting other high-dose targets from the expanded region $\left(PTV_{i}^{ext}\right)$ and then intersecting the result with the total prescription isodose volume $\left(V_{pd}\right)$, we isolate the dose volume specific to $PTV_{i}$. The modified CI is then calculated using Formula (1), substituting $V_{TV}$ with $V_{TV}^{new}$. While Figure 1 illustrates these geometric relationships using a simplified two-target model for conceptual clarity, the Boolean logic defined in Equation (2) applies generally to any number of targets (*n*) involving complex mixed interactions.

### 2.2. Patient Characteristics

This study was approved by the Institutional Ethics Committee (Approval No. JS2024-30-1). Fifteen BC cases (modified radical mastectomy) and fifteen NPC cases previously treated with VMAT at our center between July and December 2024 were retrospectively selected. BC cases included two targets (PTVsc and PTVcw) both with a prescription of 43.5 Gy in 15 fractions. PTVsc and PTVcw were partially overlapping. NPC cases included five targets (PTVp, PTVn, PTVrpn, PTV1, PTV2) with prescriptions ranging from 54.45 Gy to 69.96 Gy in 33 fractions. The large-volume target PTV1 fully included the sub-targets PTVp, PTVn and PTVrpn. Among these sub-targets, PTVp and PTVn were spatially independent, while PTVp and PTVrpn were partially overlapping. Additionally, PTVn and PTVrpn were mutually independent, and the separate nodal volume PTV2 was spatially independent from all other target volumes. Patient characteristics are summarized in Table 1 and Table 2.

### 2.3. Treatment Planning

BC plans were designed using Monaco (version 6.0; Elekta, Stockholm, Sweden) with 2 coplanar partial arcs. NPC plans were designed using Pinnacle^3^ (version 16.2; Philips Medical Systems, Milpitas, CA, USA) with 2 coplanar full arcs. The planning goal was to deliver the prescription dose to at least 95% of the PTV while minimizing OAR dose. Dose-volume calculations were carried out to obtain $TV_{PV}$, $V_{PTV}$, $V_{TV}^{old}$, and $V_{TV}^{new}$ for all targets.

### 2.4. Statistical Analysis

$V_{TV}$ and CI values were calculated using both the new and old formulas. A paired, two-tailed non-parametric Wilcoxon signed-rank test was used to compare the paired data $\left(V_{TV}^{new}\;vs.V_{TV}^{old}\mathrm{and}\;CI_{new}\;vs.CI_{old}\right)$, as the data did not strictly follow a normal distribution. A *p*-value < 0.05 was considered statistically significant. Pearson’s correlation coefficients were calculated to analyze the relationship between $CI_{new}$ and $CI_{old}$.

## 3. Results

### 3.1. Performance Evaluation of $V_{TV}$

**Figure 2:**
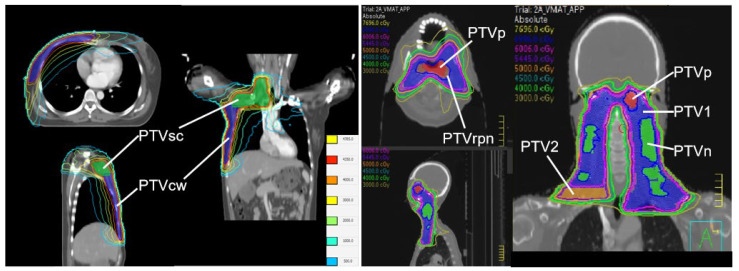
Dose distribution contour maps of a BC and an NPC plan.

Visual inspection of dose distributions confirmed good conformity for all PTVs (Figure 2). Table 3 presents the comparison between $V_{TV}^{new}$ and $V_{TV}^{old}$. For BC, $V_{TV}^{new}$ values for PTVsc and PTVcw were significantly lower than $V_{TV}^{old}$ (*p* < 0.001), indicating that the traditional formula overestimated the volume by including dose from the adjacent target. For NPC, similar significant reductions were observed for PTVp, PTVn, PTVrpn, and PTV2 (*p* < 0.001). However, for PTV1 (which encompasses other targets), the results were identical; p=1.000.

### 3.2. Performance Evaluation of CI

Table 4 compares the conformity indices. For BC, $CI_{new}$ values were significantly higher than $CI_{old}$ for both targets (*p* < 0.001). For example, mean PTVsc CI increased from 0.315 to 0.827, correcting the artificial underestimation caused by the traditional formula. For NPC, $CI_{new}$ was significantly higher for all targets except PTV1, where the values were identical.

### 3.3. Correlation Analysis

Pearson’s correlation analysis (Table 5) showed a positive correlation between $CI_{new}$ and $CI_{old}$ for PTVcw in BC (r=0.640,p<0.05) and PTVrpn in NPC (r=0.660,p<0.05). No significant correlation was found for other independent/overlapping targets, suggesting that the traditional CI often fails to capture the true conformity trend in complex multi-target scenarios.

## 4. Discussion

The CI is a critical tool for evaluating plan quality and guiding optimization. However, the geometric complexity of multi-target plans—characterized by varying prescription doses and overlapping volumes—challenges the traditional calculation methods. The standard formula proposed by van’t Riet and Paddick defines $V_{TV}$ as the total volume of the reference isodose. While accurate for single targets or complete inclusion geometries, this definition fails in partial overlap or independent relationships because it erroneously includes dose contributions from adjacent targets. This distortion leads to artificially low CI values, potentially misguiding clinical decision-making.

Previous studies have attempted to address the limitations of standard conformity indices in multi-target environments. Venkataraman et al. [33] proposed a manual “margin contour” method, creating auxiliary structures (e.g., 2 mm expansions) to physically isolate the local prescription dose for each target. Harikrishnaperumal [34] introduced a graphical analysis technique, calculating conformity within varying annular regions to identify a stable “plateau” representing the true conformity. However, these approaches often require manual contouring or iterative graphical interpretation. Salari et al. [35] utilized the standard Paddick and RTOG conformity indices to evaluate single-isocenter multi-target plans. Their approach relied on the traditional definition of $V_{TV}$ (total reference isodose volume), and they reported that these standard metrics are heavily influenced by the number of targets and dose bridging. In contrast to these methods, our proposed Generalized CI utilizes standard Boolean logic to mathematically redefine $V_{TV}$. Unlike the standard formulas used by Salari et al. [35], which include the global isodose volume, or the manual workarounds of Venkataraman et al. [33], our formula automatically subtracts non-local dose contributions. This provides a direct, calculated solution that isolates the specific target’s conformity regardless of the surrounding multi-target complexity.

Our results confirm that the new formula effectively corrects these distortions. For BC cases, the $CI_{new}$ accurately reflected the high quality of the VMAT plans visually observed. Similarly, for NPC, the method distinguished between targets requiring correction and those where the traditional formula was sufficient (PTV1). The identical results for PTV1 serve as a validation of the method, proving that the generalized formula simplifies to the traditional one when geometric conditions (complete inclusion) allow it.

The specific expansion parameters used in our generalized formula were derived from a statistical analysis of historical single-target plans at our institution. We found that an anisotropic expansion of 1.0 cm in the anterior/posterior and left/right directions and 0.5 cm in the superior/inferior (SI) direction was sufficient to fully encompass the prescription isodose volume in highly conformal VMAT plans. The 1.0 cm axial expansion accounts for the typically broader dose fall-off in the transverse plane, while the tighter 0.5 cm SI margin reflects the steeper gradients achievable in the longitudinal direction, which is critical for sparing closely stacked targets in head and neck cases. It is important to note that these values function as adaptable parameters within the generalized framework. For institutions where treatment planning priorities result in broader dose spillover (i.e., less conformal dose distributions), these expansion margins can be readily adjusted—for example, to 2.0 cm axially and 1.0 cm longitudinally—to ensure the interfering dose is fully captured.

We specifically selected BC and NPC for this analysis because they represent the two most geometrically challenging scenarios in multi-target planning. BC cases typically exhibit “small overlap” relationships, while NPC cases present a complex “mixed” environment of inclusion, independence, and intersection. Since the proposed generalized formula is based on fundamental Boolean logic capable of resolving these extreme geometric states, it is mathematically applicable to other tumor sites with similar multi-target configurations. Consequently, this method can be extended to other clinical scenarios, such as SIB treatments for rectal or prostate cancer, without requiring separate validation for every anatomical site.

This study has several limitations that should be noted. First, the study utilized a relatively limited dataset n=30. While sufficient for the mathematical proof-of-concept of the generalized formula, future studies with larger cohorts are warranted to establish clinical benchmarks. Second, all treatment plans were generated at a single institution. While this may introduce institutional planning bias regarding absolute dosimetric quality, the relative geometric relationship addressed by the formula remains valid. Third, absolute dosimetric values may vary across different TPS due to differences in calculation algorithms; however, since the proposed formula relies on standard Boolean volume operations, it can be readily implemented in any commercial TPS using native ROI algebra tools. Finally, regarding statistical analysis, while Pearson’s correlation was used to illustrate trends, future studies with larger sample sizes should utilize non-parametric correlation methods if normality assumptions are not strictly met.

## 5. Conclusions

We have introduced a generalized $V_{TV}$ calculation formula that enables the accurate assessment of conformity in multi-target radiotherapy plans. By isolating the prescription dose range of the target under analysis, the new formula eliminates the confounding influence of adjacent target doses. This method is universally applicable, yielding results identical to traditional formulas for single targets while providing corrected, accurate indices for complex multi-target geometries. Future research should aim to correlate these improved dosimetric indices with clinical outcomes, such as local tumor control and normal tissue toxicity, to establish their prognostic value.

## Figures and Tables

**Table 1:** Basic and clinical characteristics of BC patients (*n* = 15).

Gender			
Male	0		
Female	15		
	Age (years)	Volume of PTVsc (cc)	Volume of PTVcw (cc)
mean	55.1	234	405
median	53	224	376
range	41–76	168–309	278–656

**Table 2:** Basic and clinical characteristics of NPC patients (*n* = 15).

Gender						
Male	9					
Female	6					
	Age (years)	Volume of PTVp (cc)	Volume of PTVn (cc)	Volume of PTVrpn (cc)	Volume of PTV1 (cc)	Volume of PTV2 (cc)
mean	56.1	87.4	44.0	16.8	609	111
median	57	89.0	47.1	13.0	602	124
range	30–76	43.7–156	12.7–85.6	3.82–60.2	428–797	10.6–207

**Table 3:** Comparison of $V_{TV}$ (cc) between new and old formulas.

Disease	Target	$V_{TV}^{new}$ (Mean ± SD)	$V_{TV}^{old}$ (Mean ± SD)	Z-Score	*p*-Value
BC	PTVcw	449 ± 111	694 ± 135	−3.408	0.001
	PTVsc	261 ± 39	694 ± 135	−3.408	0.001
NPC	PTVp	92.2 ± 36.5	158 ± 41	−3.408	0.001
	PTVn	52.5 ± 24.5	158 ± 41	−3.408	0.001
	PTVrpn	19.6 ± 17.4	158 ± 41	−3.408	0.001
	PTV1	610 ± 96	610 ± 96	0	1
	PTV2	119 ± 53	885 ± 113	−3.408	0.001

Note: *p*-values were calculated using the Wilcoxon signed-rank test. $V_{TV}^{new}$: Volume calculated by generalized formula; $V_{TV}^{old}$: Volume calculated by traditional formula.

**Table 4:** Comparison of CI between new and old formulas.

Disease	Target	$CI_{new}$ (Mean ± SD)	$CI_{old}$ (Mean ± SD)	Z-Score	*p*-Value
BC	PTVcw	0.800 ± 0.029	0.515 ± 0.048	−3.408	0.001
	PTVsc	0.827 ± 0.034	0.315 ± 0.044	−3.408	0.001
NPC	PTVp	0.853 ± 0.034	0.495 ± 0.142	−3.408	0.001
	PTVn	0.723 ± 0.046	0.241 ± 0.096	−3.408	0.001
	PTVrpn	0.735 ± 0.086	0.096 ± 0.090	−3.408	0.001
	PTV1	0.875 ± 0.015	0.875 ± 0.015	0	1
	PTV2	0.824 ± 0.032	0.113 ± 0.050	−3.408	0.001

Note: *p*-values were calculated using the Wilcoxon signed-rank test. $CI_{new}$: CI calculated by generalized formula; $CI_{old}$: CI calculated by traditional formula.

**Table 5:** Correlation analysis between $CI_{new}$ and $CI_{old}$.

	BC		NPC				
	PTVcw	PTVsc	PTVp	PTVn	PTVrpn	PTV1	PTV2
Correlation Coefficient (*r*)	0.640	0.310	−0.125	0.199	0.660	1.000	0.168
*p*-value	0.010	0.261	0.658	0.476	0.007	0.000	0.549

Note: *r* = Pearson’s correlation coefficient.

## Data Availability

The data presented in this study are available upon request from the corresponding author. The data are not publicly available due to privacy and ethical restrictions regarding patient information.
